# Exploration of Flexible Pes Planus as a Potential Risk Factor for Cardiac Pathologies in Pediatric Cases

**DOI:** 10.7759/cureus.46139

**Published:** 2023-09-28

**Authors:** Yavuz Selim Karatekin, Harun Altinayak, Şeyma Karatekin, Hüseyin Karadağ, Emre Usta, Bedri Karaismailoğlu, Ali Seker

**Affiliations:** 1 Orthopedics and Traumatology, Samsun Training and Research Hospital, Samsun, TUR; 2 Orthopaedics and Traumatology, Samsun Training and Research Hospital, Samsun, TUR; 3 Pediatrics, Faculty of Medicine, Samsun University, Samsun, TUR; 4 Pediatric Cardiology, Dr. Siyami Ersek Thoracic and Cardiovascular Surgery Training and Research Hospital, University of Health Sciences, Istanbul, TUR; 5 Pediatric Cardiology, Faculty of Medicine, Samsun University, Samsun, TUR; 6 Orthopaedics and Traumatology, Cerrahpasa Medical School, Istanbul University-Cerrahpasa, Istanbul, TUR; 7 Orthopaedics and Traumatology, Cerrahpasa Medical Faculty, Istanbul University-Cerrahpasa, Istanbul, TUR

**Keywords:** valvular heart disease, heart failure, mitral valve prolapse, hypermobility, echocardiography, cardiac pathology, flat foot, pes planus

## Abstract

Objective: The aim of this study is to investigate the relationship between flexible pes planus (FPP) and cardiac pathologies in pediatric patients, with a particular emphasis on hypermobility status.

Methods: Between January and June 2022, a retrospective screening was conducted on a total of 68 patients aged between 6 and 18 years who had been diagnosed with FPP. Eight patients were excluded from the study due to suspicion of connective tissue or neuromuscular diseases following systemic examinations conducted by a pediatric specialist. The included 60 patients underwent comprehensive cardiac examinations conducted by pediatric cardiology specialists and were evaluated through echocardiography (ECHO) as part of the study. Simultaneously, a control group of healthy individuals aged 6 to 18, without a diagnosis of pes planus (PP), was selected from those who applied for health reports for the purpose of obtaining sports licenses. Patients diagnosed with FPP were compared to a control group in terms of the presence of cardiac pathology. Furthermore, the Beighton Hypermobility Scores (BHSs) of patients diagnosed with FPP were compared based on the presence of cardiac pathology.

Results: A total of 60 patients (40 males, 20 females) diagnosed with FPP were included in the study, and a control group consisting of 453 healthy individuals (287 males, 166 females) was selected. The incidence of cardiac pathology in patients diagnosed with FPP (23.3%) was found to be significantly higher compared to the control group (7%) (p <0.01). The risk of cardiac pathology in patients diagnosed with FPP was determined to be four times higher compared to the control group (Odds ratio: 4 (1.993-8.046), p<0.01). Among patients with FPP, individuals who were found to have cardiac pathology had statistically significantly higher average BHSs compared to those without cardiac pathology (p: 0.043).

Conclusions: Our study suggested that there may be a significant relationship between FPP and high cardiac pathology risk in pediatric patients. We observed a significant increase in the incidence of cardiac pathologies among patients diagnosed with FPP. Additionally, the presence of higher hypermobility scores among patients diagnosed with FPP suggests a potential connection between the two. In the evaluation of FPP in the pediatric population, it should be considered as a potential risk factor for cardiac pathologies and its potential association with hypermobility.

## Introduction

Pes planus (PP), typically manifests as a pathology characterized by the valgus position of the hindfoot and the collapse of the medial longitudinal arch (MLA) [1]. PP, which is among the most common reasons for orthopedic clinic visits, is generally functional and painless [2]. However, parents often have concerns about the potential for pain and disability in later years. PP tends to decrease with age. Its prevalence in children is over 70% in the first four to six years of life, but it has been reported to decrease to around 9% after the age of six [3,4].

Following the examinations, PP is assessed within two primary categories, flexible and rigid, based on its characteristics. Rigid PP typically harbors an underlying pathology, necessitating further investigation into these pathologies. Instances of such pathologies may encompass neuromuscular disorders, cerebral palsy, congenital anomalies, and conditions such as tarsal coalition [1,2]. On the other hand, in the case of flexible pes planus (FPP), it is believed that ligament laxity plays a significant role. In addition, it should be noted that hypermobility may be a component of connective tissue disorders such as Marfan syndrome and Ehler-Danlos syndrome [5]. In such conditions, it is known that systemic issues, including cardiac pathologies especially, are present. During the examination, adopting a systematic approach is important to ensure that conditions like these are not overlooked.

Hypermobility refers to the condition that the individual has more than normal range of motion of the synovial joints and may accompany some connective tissue diseases. If an individual presents with additional symptoms alongside hypermobility, it is defined as hypermobility syndrome [6]. Hypermobility is assessed using the Beighton score, while the Brighton criteria are employed to diagnose hypermobility syndrome [6]. As it is well-established, individuals with hypermobility syndromes may present with concomitant heart conditions, with mitral valve prolapse (MVP) being a noteworthy example [7]. In addition, it has been demonstrated that as the degree of FPP increases, the risk of concomitant MVP also increases [8]. Furthermore, in connective tissue disorders characterized by hypermobility such as Marfan syndrome and Ehler-Danlos syndrome, cardiac pathologies can also be encountered [9,10]. 

Our hypothesis is that in patients aged six and above diagnosed with FPP, hypermobility may be present even in the absence of hypermobility syndrome or connective tissue disorders, and cardiac pathologies may be observed in these patients. The purpose of this study is to investigate the relationship between FPP and cardiac pathologies by examining them through the echocardiography (ECHO) method, and to compare the presence of cardiac pathologies between FPP patients and healthy individuals. Furthermore, to assess the relationship between hypermobility and the presence of cardiac pathology, the comparison of Beighton Hypermobility Scores (BHSs) between patients with and without cardiac pathology has also been targeted in this study.

## Materials and methods

After obtaining Institutional Review Board approval (SÜKAEK-2023 5/5), a retrospective examination was conducted on patients aged six to 18 diagnosed with FPP in the orthopedics and traumatology outpatient clinic between January and June 2022. Patients with detailed clinical notes and direct radiographs on the PACS system were included in the study. Patients with a history of prior foot or ankle fractures, lower limb deformities, rigid PP, tarsal coalition, or hindfoot movement restrictions were excluded from the study. A total of 68 patients were included in the study following examination and assessment. Demographic information such as age, gender, and BHS were recorded for the patients. Patients were directed to a pediatric specialist (Ş.K.) for supplementary assessment of connective tissue disorders and a systemic examination. Eight patients with suspected connective tissue or neuromuscular diseases were excluded from the study. The remaining 60 patients underwent general cardiac examinations conducted by pediatric cardiology specialists (E.U. and H.K.) in terms of cardiac pathologies, and they were assessed using ECHO.

Between January and June 2022, a control group of healthy individuals aged six to 18, without a diagnosis of PP, was selected from those who applied for health reports for the purpose of obtaining sports licenses. (Routine echocardiographic assessments are conducted as part of the health examination for sports license applications). Clinical examinations, demographic data, and ECHO results were retrospectively reviewed. Individuals with systemic diseases, connective tissue diseases, and neuromuscular diseases were excluded from the control group. Data from a total of 453 individuals were accessed and recorded. Patients diagnosed with FPP were compared to healthy individuals in the control group in terms of the presence of cardiac pathology. Furthermore, the BHS of patients diagnosed with FPP were compared based on the presence of cardiac pathology.

Diagnosis of FPP on examination

· The observation of MLA collapse and hindfoot valgus position during weight-bearing

· The determination of the return of MLA to normal through the Jack's test or rising onto tiptoes

· The normal range of motion in the subtalar joint

Echocardiographic examinations method

Echocardiographic examinations were performed using a Vivid 7 device (GE Medical Systems, Horton, Norway) equipped with a 1.3-3.6 MHz multifrequency transducer. The ECHO protocol involved comprehensive cardiac assessments conducted simultaneously by at least two experienced cardiologists to minimize measurement errors. Skilled cardiologists interpreted the echocardiograms. Measurements were taken for proximal ascending aorta diameter (PAO), left ventricular end-diastolic dimension (LVEDd), interventricular septal wall thickness (IVST), left ventricular posterior wall thickness (LVPWT), and left ventricular ejection fraction (LVEF) following the guidelines of the American Society of Echocardiography [11]. LVEDd, IVST, and LVPWT were measured using M-mode ECHO in the parasternal long-axis view. All detectable regurgitations were identified and categorized as mild, moderate, or severe in accordance with the recommendations of the American Society of Echocardiography [12]. Echocardiographic abnormalities were defined as cardiac chamber or major artery dimensions exceeding the normal range for the patient's peers (Z-score > 2.0), mild to moderate valve regurgitation, and other potential echocardiographic irregularities [13]. The criteria used to define pathological mitral regurgitation (MR) include a color Doppler jet with a length exceeding 1 cm. Demonstrating the color Doppler jet in at least two different imaging planes. The continuous presence of the pulsed or continuous wave Doppler signal throughout the systolic phase. The criteria used to define pathological aortic regurgitation (AR) include was when it was observed in two views and present in at least one view, with a jet length of ≥1 cm, and a pan-diastolic jet present in at least one envelope.

Statistical analysis

Statistical analysis was performed using the Statistical Package for Social Sciences software version 21 (SPSS Inc., IBM, NY, USA). Categorical variables were given with frequency and percentage; continuous numerical variables were given with median. The chi-square test was used to compare frequencies. The comparison of Beighton scores based on the cardiac pathologies of the patients and the comparison of ages between the groups were analyzed using the Mann-Whitney U test in this study. A p-value below 0.005 was considered statistically significant.

## Results

A total of 60 patients (40 male, 20 female) were included in the study with a diagnosis of FPP, and 453 healthy individuals (287 male, 166 female) were selected as the control group. No significant differences were observed in the comparison of age averages and gender distribution between patients diagnosed with FPP and the control group (p-values were 0.071 and 0.616, respectively) (Table 1).

**Table 1:** The comparison of groups by gender and ages For gender comparison, the chi-square test was utilized, whereas the Mann-Whitney U test was employed for age comparison. FPP: Flexible pes planus

Group	Gender		Total	P value	Age		P value
	Female	Male			Mean	Std. Deviation	
FPP	20	40	60	0.616	9.90	3.348	0.071
Control Group	166	287	453		10.92	2.884	
Total	186	327	513				

The incidence of cardiac pathology in patients diagnosed with FPP (23.3%) was significantly higher compared to the control group (7%), and the likelihood of having cardiac pathology in FPP patients was determined to be four times higher than that of the control group (P<0.01) (Odds ratio: 4) (Table 2).

**Table 2:** Frequency of cardiac pathology in PP patients and the control group Chi-Square Tests FPP: Flexible pes planus; PP: Pes planus

	Control Group		FPP		P value	Odds Ratio	95% Confidence Interval	
Cardiac Pathology	Frequency	Percent	Frequency	Percent			Lower	Upper
Negative	421	93	46	76.7	P<0.01	4	1.993	8.046
Positive	32	7	14	23.3				
Total	453	100	60	100				

Cardiac pathologies detected in patients diagnosed with FPP are presented in Table 3, while cardiac pathologies in the control group are presented in Table 4.

**Table 3:** Cardiac pathologies detected in PP patients MVP: Mitral valve prolapse, MR: Mitral regurgitation, VSD: Ventricular septal defect, AR: Aortic regurgitation; PP: Pes planus

Patient Number	Age	Gender	Cadiac Pathology	Patient Number	Age	Gender	Cardiac Pathology
1	10	M	MVP	8	10	F	MR
2	7	M	VSD, MVP, MR	9	9	M	AR
3	7	F	MR	10	12	F	MR
4	16	M	MR	11	15	M	MR
5	9	M	Sinus of Valsalva Aneurysm, AR	12	7	F	MR
6	11	F	MR	13	8	M	MVP, MR
7	11	F	MR	14	10	M	MVP

**Table 4:** Cardiac pathologies detected in the control group MVP: Mitral valve prolapse, MR: Mitral regurgitation, ASD: Atrial septal defect, AR: Aortic regurgitation, PS: Pulmonary stenosis

Patient Number	Age	Gender	Cadiac Pathology	Patient Number	Age	Gender	Cardiac Pathology
1	15	F	AR	17	8	F	MR
2	17	M	MR, ASD	18	9	F	MR, MVP, ASD
3	17	F	MVP, MR	19	10	M	AR
4	15	M	MR, ASD	20	9	F	MVP, MR
5	11	F	MR	21	11	F	Left Ventricular Hypertrophy
6	14	F	AR	22	11	M	ASD
7	14	M	MR	23	7	M	Bicuspid Aortic Valve , AR, Ascending Aortic Dilation
8	14	F	ASD	24	9	M	Bicuspid Aortic Valve
9	10	F	MR	25	10	M	MR
10	13	F	ASD	26	8	M	MR
11	13	M	MR	27	7	F	MR
12	10	M	MR	28	12	M	MR, MVP, Ascending Aortic Dilation
13	13	M	AR	29	8	F	MR
14	9	F	MR, AR, ASD, Left Ventricular Systolic Dysfunction	30	10	M	MR
15	7	M	ASD	31	10	M	Bicuspid Aortic Valve, AR,
16	15	M	MR	32	8	M	PS

Various cardiovascular abnormalities were identified among patients diagnosed with FPP in the clinical sample. In this context, four patients had MVP, 10 patients had mild MR, one patient had a small-sized ventricular septal defect (VSD), two patients had mild AR, and one patient had a Sinus of Valsalva aneurysm. In the control group, four patients had MVP, 19 patients had mild MR, eight patients had atrial septal defect (ASD), seven patients had mild AR, one patient had left ventricular systolic dysfunction, one patient had pulmonary stenosis (PS), one patient had left ventricular hypertrophy, three patients had bicuspid aortic valve, and two patients had ascending aortic dilation. The most frequently identified cardiac pathology in both groups is mild MR (Tables 5, 6).

**Table 5:** Frequencies of cardiac pathologies detected in PP patients MVP: Mitral valve prolapse, MR: Mitral regurgitation, VSD: Ventricular septal defect, AR: Aortic regurgitation; PP: Pes planus

Cardiac Pathology	Frequency	Percent
MVP	4	20
MR (Mild)	10	50
VSD (Small Size)	1	5
AR (Mild)	2	10
Sinus of Valsalva Aneurysm	1	15
Total	20	100

**Table 6:** Frequencies of cardiac pathologies detected in the control group MVP: Mitral valve prolapse, MR: Mitral regurgitation, ASD: Atrial septal defect, AR: Aortic regurgitation, PS: Pulmonary stenosis

Cardiac Pathology	Frequency	Percent
MVP	4	8.6
MR (Mild)	19	41.3
ASD	8	17.3
AR (Mild)	7	15.2
Left Ventricular Systolic Dysfunction	1	2.1
PS (Mild)	1	2.1
Left Ventricular Hypertrophy	1	2.1
Bicuspid Aortic Valve	3	6.5
Ascending Aortic Dilation	2	4.3
Total	46	100.0

Among patients with FPP in whom cardiac pathology was detected, the average BHS scores were statistically significantly higher compared to those without cardiac pathology (p: 0.043) (Table 7).

**Table 7:** Comparison of Beighton scores in PP patients regarding the presence of cardiac pathology Mann-Whitney U Test PP: Pes planus, BHS: Beighton Hypermobility Score

	Cardiac Pathology	N	Mean	Std. Deviation	P Value
BHS	Negative	46	3.52	1.426	0.043
	Positive	14	4.57	1.910	

## Discussion

The most prominent finding of our study is that in the pediatric patient group diagnosed with FPP without any connective tissue or other systemic diseases, the likelihood of cardiac pathology is four times higher compared to healthy individuals in the same age range. In this context, it may be necessary to consider the risk of cardiac pathology, particularly when evaluating patients diagnosed with FPP. Furthermore, it has been observed that patients with FPP who have been diagnosed with cardiac pathology are more hypermobile compared to those without cardiac pathology. This situation highlights the need for a more detailed investigation into the potential relationship between hypermobility and cardiac pathology.

PP is defined as the absence of the MLA and a valgus position of the hindfoot [2]. While it is observable in all infants at birth, during the preschool period, the development of the MLA occurs as the muscles and ligaments in the foot strengthen, leading to a decrease in the frequency of PP [1,14]. The structures that prevent the collapse of the MLA include bones and ligamentous structures. Huang et al. stated in their biomechanical study that the most important structures contributing to the formation of the MLA are the plantar fascia, plantar ligaments, and the spring ligament [15]. Various studies in the literature emphasize that the most critical structures preventing the collapse of the MLA are ligaments and the surrounding intrinsic muscles [16,17]. In light of all this data, it has been emphasized that ligament laxity plays a prominent role among the causes of FPP. Furthermore, although it has been associated with conditions such as Marfan syndrome, Fragile X syndrome, and cerebral palsy, FPP is generally described as idiopathic [18]. Although there are different opinions in the literature, it is also known that connective tissue diseases such as Marfan and Ehlers-Danlos can be accompanied by cardiac pathologies [19]. Additionally, it is also known that cardiac pathologies can accompany thoracic and spinal skeletal anomalies [20-22]. AbdelMassih et al., emphasized in their study that the risk of MVP increases with the degree of FPP [8]. In our study, it was found that the risk of cardiac pathology in patients diagnosed with FPP is higher compared to normal individuals, similar to what is observed in connective tissue diseases, and a result consistent with the literature was obtained. This finding underscores the need for a more in-depth understanding of the potential relationship between FPP and cardiac pathologies. However, a more comprehensive understanding of this relationship requires further research. Previous studies have shown that the prevalence of MVP in screening with ECHO in the general population varies between 1% and 7%, while MR varies between 1% and 3% [23,24]. The concordance of cardiac pathology rates in our control group with the literature demonstrates that an appropriate control group selection has been made in our study.

The condition characterized by the presence of symptoms alongside hypermobility is termed as Joint Hypermobility Syndrome. In our study, isolated FPP patients without any symptoms and without a diagnosis of systemic connective tissue disease were examined, and hypermobility was assessed using the BHS. Malfait et al. emphasized in their study that there may be a relationship between collagen chain deficiency and hypermobility, and due to the risk of complications, patients should be evaluated for cardiac concerns [25]. There are numerous publications in the literature that emphasize the association between hypermobility and cardiac pathologies [26,27]. In the study conducted by Grahame et al., patients with hypermobility were assessed using ECHO, and it was observed that the rate of cardiac pathology was higher compared to the patients in the control group [28]. But there are contrasting views in the literature. In Mishra et al.'s study, it was stated that there was no relationship between joint hypermobility syndrome and cardiac pathologies, and no correlation was found between the BHS and cardiac pathologies [29]. Although there may not be a complete consensus, it is generally accepted that there is a relationship between hypermobility and cardiac pathologies. According to the study conducted by Yazıcı et al., it was emphasized that there is a relationship between the BHS and cardiac pathologies [7]. In our study, when comparing patients diagnosed with FPP, those with cardiac pathologies had higher average BHS, which is consistent with the literature.

This study highlights the importance of evaluating cardiac health in individuals diagnosed with FPP. Furthermore, the higher BHS in FPP-diagnosed patients with cardiac pathology indirectly support the relationship between cardiac pathology and connective tissue. Future comprehensive studies may contribute to elucidating the mechanism of this relationship and gaining a better understanding of the risk of cardiac pathology. As far as we are aware, we have not encountered another multidisciplinary paper that examines the relationship between FPP and all cardiac pathologies. This study has certain limitations.

The most significant limitation is that genetic testing related to connective tissue diseases was not conducted for all patients. The second significant limitation is that ECHO results may vary depending on the operator. Another important limitation is that the study covered a six-month period and was not conducted throughout the entire year.

## Conclusions

Our study suggested that there may be a significant relationship between FPP and high cardiac pathology risk in pediatric patients. We observed a significant increase in the incidence of cardiac pathologies among patients diagnosed with FPP. Additionally, the presence of higher hypermobility scores among patients diagnosed with FPP suggests a potential connection between the two. In the evaluation of FPP in the pediatric population, it should be considered as a potential risk factor for cardiac pathologies and its potential association with hypermobility.
